# Using a Syndemics Framework to Understand How Substance Use Contributes to Morbidity and Mortality among People Living with HIV in Africa: A Call to Action

**DOI:** 10.3390/ijerph19031097

**Published:** 2022-01-19

**Authors:** Emmanuel Peprah, Bronwyn Myers, Andre-Pascal Kengne, Nasheeta Peer, Omar El-Shahawy, Temitope Ojo, Barbara Mukasa, Oliver Ezechi, Juliet Iwelunmor, Nessa Ryan, Fatoumata Sakho, John Patena, Joyce Gyamfi

**Affiliations:** 1 Global Health Program, New York University School of Global Public Health, New York, NY 10003, USA; elshao01@nyu.edu (O.E.-S.); to790@nyu.edu (T.O.); ryann01@nyu.edu (N.R.); fs2291@nyu.edu (F.S.); john.patena@nyu.edu (J.P.); gyamfj01@nyu.edu (J.G.); 2 Curtin enAble Institute, Faculty of Health Sciences, Curtin University, Perth, WA 6845, Australia; Bronwyn.Myers@mrc.ac.za; 3 Alcohol, Tobacco and Other Drug Research Unit, South African Medical Research Council, Cape Town 7505, South Africa; 4 Division of Addiction Psychiatry, Department of Psychiatry and Mental Health, University of Cape Town, Cape Town 7925, South Africa; 5 Non-Communicable Diseases Research Unit, South African Medical Research Council, Cape Town 7505, South Africa; andre.kengne@mrc.ac.za (A.-P.K.); nasheeta.peer@mrc.ac.za (N.P.); 6 Department of Medicine, Faculty of Health Sciences, University of Cape Town, Cape Town 7925, South Africa; 7 Department of Population Health, New York University Grossman School of Medicine, New York, NY 10016, USA; 8 Center for the Prevention of Heart Disease, John Hopkins Hospital, Baltimore, MD 21287, USA; 9 Mildmay Uganda, Kampala P.O. Box 24985, Uganda; barbara.mukasa@mildmay.or.ug; 10 Nigerian Institute of Medical Research, Yaba, Lagos 101245, Nigeria; oezechi@yahoo.co.uk; 11 College for Public Health and Social Justice, Department of Behavioral Science and Health Education, Saint Louis University, St. Louis, MO 63104, USA; juliet.iwelunmor@slu.edu

**Keywords:** substance use, people living with HIV (PWH), Africa, syndemics

## Abstract

Substance use is increasing throughout Africa, with the prevalence of alcohol, tobacco, cannabis, and other substance use varying regionally. Concurrently, sub-Saharan Africa bears the world’s largest HIV burden, with 71% of people living with HIV (PWH) living in Africa. Problematic alcohol, tobacco, and other substance use among PWH is associated with multiple vulnerabilities comprising complex behavioral, physiological, and psychological pathways that include high-risk behaviors (e.g., sexual risk-taking), HIV disease progression, and mental health problems, all of which contribute to nonadherence to antiretroviral therapy. Physiologically, severe substance use disorders are associated with increased levels of biological markers of inflammation; these, in turn, are linked to increased mortality among PWH. The biological mechanisms that underlie the increased risk of substance use among PWH remain unclear. Moreover, the biobehavioral mechanisms by which substance use contributes to adverse health outcomes are understudied in low- and middle-income countries (LMIC). Syndemic approaches to understanding the co-occurrence of substance use and HIV have largely been limited to high-income countries. We propose a syndemic coupling conceptual model to disentangle substance use from vulnerabilities to elucidate underlying disease risk for PWH. This interventionist perspective enables assessment of biobehavioral mechanisms and identifies malleable targets of intervention.

## 1. Substance Use Varies by African Regions 

Global Burden of Disease (GBD) data indicate that substance use (SU) varies by African region, with the highest age-standardized prevalence of alcohol, cannabis, and other substance use in the Eastern region of sub-Saharan Africa (SSA) (1611 per 100,000), followed by the Southern (1515 per 100,000), Central (1413 per 100,000), and Western (1168 per 100,000) regions (Table 1) [1]. People living with HIV (PWH) experience higher levels of mental health concerns, including mood disorders, anxiety disorders, and substance use disorders (SUD); these disorders lead to poorer adherence to antiretroviral therapy (ART) [2,3]. Modelling of these data indicates that significant population growth and ageing will result in an estimated 130% increase in the burden of mental health issues related to SUD by 2050, which could reinforce nonadherence to ART [4,5]. Moreover, Africa contains approximately 12% of the global population, yet 71% of the global PWH population reside on the continent. Globally, approximately 6000 incident cases of HIV occur daily, of which two-thirds are in SSA, with young women aged 15–24 bearing a disproportionate burden [6].

SU is used in this commentary to refer to general substance use, particularly when cited information does not specify whether it is substance use or clinically diagnosed substance use disorder. The DSM-5 defines SUD by patterns of symptoms and SU, with specific criteria required for SUD diagnosis. Despite its growing significance, there is a dearth of data on SU and SUD in SSA. Lack of routine collection of SU information in HIV services [7,8] represents a missed opportunity for programmatic data. When data are collected, stigma leads to under-reporting [9]. Nonetheless, existing data suggest that SU is increasing in Africa, particularly among PWH [9,10]. Harmful patterns of SU are associated with increased sexual risk for HIV [11,12]. Among PWH, SU is associated with adverse HIV treatment outcomes, including decreased ART initiation, suboptimal adherence to ART [13,14], and early mortality [15,16]. The limited data on harmful patterns of alcohol consumption in Africa, specifically heavy episodic drinking (HED; sometimes termed binge drinking), highlight HED as a risk factor for risky sexual behavior, HIV infection, and poor engagement in each step of the HIV care cascade [14,17,18,19,20].

Notably, alcohol is the most prevalent substance used in all four SSA regions (Table 1) and accounts for more disability-adjusted life years (DALYs) lost than other substances (Table 2). Opioids are the next most prevalent substance, followed by cannabis. The most recent World Drug Report (circa 2021) notes increasing prevalence of opioids in Africa [21]. Tramadol is a prescription opioid of choice in Africa; there was a 14-fold increase in tramadol seizures in Central, West, and North Africa between 2013 and 2017 [21]. Moreover, in the past two decades, there has been an increase in the African illicit drug market [20]. East and Southern African countries (e.g., Ethiopia, Kenya, Tanzania, and South Africa) and Nigeria serve as transit hubs for Afghani and Pakistani heroin and Asian synthetic drugs [21,22,23]. West African countries (e.g., Nigeria) are transit hubs for South American cocaine on its way to Europe and the United States [23]. South Africa also produces and serves as a transit hub for amphetamine-type stimulants [23,24]. According to Emerson and Solomon, “Africa’s role as a significant transit hub in the global drug trade … is leading to increased domestic drug use and creating a burgeoning health challenge” [23,25,26]. Existing data demonstrate substantial regional variation within SSA (Table 2) [1].

The United Nations Office of Drugs and Crime (UNODC) predicted that Africa will see the largest increase in the number of people who use drugs in the next decade among all world regions [27]. The projected 40% increase from 60 million in 2018 to 86 million by 2030 is unique to Africa [27]. Globally, the number of people using drugs is projected to rise by 11% by 2030 because of demographic transitions [27]. Although, there is an expected increase in the number of people who use drugs globally, a unique situation exists in Africa due to the confluence of several trends, including a younger population and higher drug use among young people than among older people [27]. Moreover, the population of Africa is projected to grow more rapidly than other world regions because of concentrations of poverty, lack of access to resources, and large demographic transitions [27]. 

This commentary explores three case studies for understanding how SU contributes to morbidity and mortality of PWH in Africa using available data on SU from Uganda, South Africa, and Nigeria. All three countries are in areas that are transit hubs for various substances and have large populations of PWH, but each has a unique context for SU. In Uganda, we examine SU among PWH based on regional data primarily from adjacent countries. Next, we explore the South African context, where there are significant data on SU on PWH and then survey Nigeria to present preliminary unpublished data from our cohort. We conclude with a discussion on how a syndemics framework can explicate the under-studied area of SU among PWH at risk of various co-morbid communicable (e.g., TB) and non-communicable (cardiovascular disease, COPD, and diabetes) diseases. 

## 2. Substance Use and HIV Prevalence among Populations in Uganda

There are approximately 1.3 million PWH in Uganda [28]; however, recent data on SU among PWH is scant. Among the general population aged 15 years and above in Uganda, prevalence of SUD differs among males (3.35%) and females (0.36%), with less difference in reported SUDs by sex (males 0.09%; females 0.03%) [29]. Ten per 100,000 inhabitants report injection drug use [29], and males are more likely than females to use tobacco (OR 5.51; 95% CI: 3.81–7.95) [30]. Moreover, the GBD study indicates that SU is most prevalent in Eastern Africa primarily using data from Kenya and Tanzania; Uganda contributes no current SU prevalence data because national statistics on SU are not available. 

Nevertheless, SU appears to be increasing in Uganda, especially among young men, although a marked lack of longitudinal studies limits the accuracy of prevalence statistics. According to a Uganda police crime report, reported narcotics cases increased by 4.0% from 2740 reported cases in 2014 to 2854 in 2017 [31]. In a cross-sectional survey conducted among PWH in Kampala, 33.0% reported using any alcohol, 18.6% reported misusing alcohol, and 5.2% reported drinking hazardous amounts of alcohol [17]. These data support WHO statistics that indicate that alcohol consumption in Uganda is significantly higher compared to other countries in the region [32]. 

Although there are limited studies in Uganda, one rapid situational assessment of people who inject drugs (PWID) in Kampala and Mbale found that 72% reported injecting heroin and 20% reported injecting cocaine [33]. There is a lack of data for SU and SUD in Uganda among both the general population and PWH. Given that many studies in South Africa have found that PWH face greater stigma than the general population and have higher utilization of various substances, robust studies are needed to characterize this population. Current data from adjoining countries including Kenya and Tanzania are not sufficient to elucidate the syndemic factors that contribute to SU and SUD in Ugandan populations and also to drug use. Robust research is needed to fill this significant knowledge gap and inform policy and the implementation of evidence-based interventions to address the needs of PWH in Uganda.

## 3. Substance Use and HIV Prevalence among South African Populations

Globally, South Africa remains the country with the highest prevalence of HIV infection, with an estimated 8.2 million PWH [34]. SU is also highly prevalent: the age-standardized prevalence of SU, including alcohol, tobacco, heroin, and amphetamine-type stimulants, is the second highest in SSA [1]. Nationally, an estimated 13.3% of the adult population meet diagnostic criteria for a lifetime SUD (any type excluding tobacco), but significant interprovincial differences exist, with rates increasing to 20.6% for the Western Cape province. Although recent years have seen increased use of cannabis, opiates, and amphetamine-type stimulants (e.g., methamphetamine), alcohol remains the most commonly used substance in South Africa [35,36,37,38,39]. 

Although more than half of the adult population abstain from using alcohol, the volume of per capita alcohol consumption among people who do drink is among the highest in the world. More specifically, HED is the most common pattern of alcohol use among people who drink [32,40]. Defined as the consumption of five or more standard drinks or 60 g of absolute alcohol during a single occasion [28], this pattern of drinking confers high levels of risk for alcohol-related harms. Notably, almost 30% of people reporting lifetime drinking in a nationally representative household survey met diagnostic criteria for a lifetime moderate or severe alcohol use disorder [36]. Similar rates of problematic alcohol use have been reported among PWH on ART; various studies report that 40% to 63% of PWH on ART who disclose drinking alcohol, report HED [41,42,43,44,45,46].

Despite comprehensive legislation against tobacco in place for two decades, smoking rates remain high among South African adults: almost 20% smoke tobacco [47,48,49]. Rates of current tobacco use are significantly higher among men than women and vary by age and ethnicity [50]. Similar smoking prevalence rates have been reported among PWH attending HIV services [50,51], and smoking often co-occurs with HED.

Community-based data on SU in South Africa are limited but suggest a growing problem. For example, a 2011 Youth Risk Behavior Survey found that 12.7% of high school students had ever used cannabis, 11.5% had used inhalants, 5.5% had used methamphetamine, 5.4% had used Mandrax (methaqualone), 5.3% had used heroin, and 4.9% had used cocaine [52]. Similarly, a 2012 population-based study among adults aged 15 and older found that the past 3-month prevalence was 4.0% for cannabis, 0.4% for sedatives, 0.3% for cocaine, 0.3% for amphetamine, 0.3% for opiates, 0.2% for inhalants, and 0.1% for hallucinogens [53]. More recently, a 2017 national study estimated the prevalence of any drug use in the last 3 months at 8.6% of the adult population [54], largely driven by cannabis use. These are likely to be underestimates, given the illicit nature of the behavior and concerns about stigma. Meanwhile, treatment admission data from six regions showed increasing treatment demand [39]. Fewer studies have investigated the prevalence of illicit drug use among PWH. An exception is Kader et al., who reported a prevalence of 13% for drug problems among 1503 HIV patients attending eight HIV clinics across Cape Town [41]. These clinic-based studies are unlikely to capture the full picture, as PWH who use illicit substances often do not initiate ART or remain engaged in care, largely due to concerns about stigma from health care providers. As such, the frequency of use of these substances among PWH who are not engaged in HIV care remains largely unknown [55,56]. 

## 4. Substance Use and HIV Prevalence among Populations in Nigeria

SU is a significant problem in Nigeria. In 2017, the UNODC published a comprehensive drug report for Nigeria. It estimated past-year prevalence of drug use at 14.4% among those aged 15 to 64 years (approximately 14.3 million people) [21]. The most prevalent drug used in Nigeria was cannabis (10.8%), followed by opioids, primarily tramadol (4.7%), cough syrups (2.4%), tranquilizers/sedatives (0.5%), ecstasy (0.3%), solvents and inhalants (0.3%), amphetamines (0.2%), cocaine (0.1%), and hallucinogens (0.03%). Polysubstance use was common in the general population, and high-risk users were defined as those who had used opioids, crack/cocaine, or amphetamines at least five times in the past month. Numerous reasons are provided for their use among the population including as a coping mechanism for stress [57].

To better characterize the SU among PWH, we conducted a cross-sectional study to examine trends in SU and identify correlates of SU among a sample of 24,488 PWH who attended the Nigerian Institute of Medical Research Center for HIV/AIDS treatment (unpublished data) between 2004 and 2018. Overall, there was a 26% increase in the prevalence of SU among PWH in this sample over the 14-year period. Notably, there was a 6% increase in self-reported opioid use from 2004 (0%) to 2018 (6%), and an estimated 3768 (15%) of PWH reported the use of at least one substance. Longitudinal surveillance of SU behaviors is needed to monitor trends and inform prevention interventions for PWH in Nigeria. 

## 5. Health Implications and Outcomes of Substance Use among People Living with HIV

SU and SUD are significant contributors to negative health outcomes in the general population; among PWH, SU, and SUD increase risk of negative outcomes and mortality [58,59,60]. Cohort studies of PWH with SU/SUD in the United States have found higher mortality among PWH who use substances than among those who do not use substances [61]. Emerging research has shown that problematic alcohol and other SU is associated with HIV disease progression and a lack of adherence to ART among PWH in SSA [62,63] (Table 2). Evidence suggests both behavioral and biologic pathways to rapid disease progression among PWH who also have SUD. Apart from the indirect effects of SU on disease progression via poor adherence, research suggests that severe SUD is associated with increases in biological markers of inflammation and negative health outcomes [64,65,66]. This is an important area of inquiry, given the research showing the increased risk of mortality among PWH, who have elevated biomarkers indicative of inflammation (i.e., IL-6, sCD14, and D-dimer) compared to HIV-negative individuals [67]. Studies of heavy alcohol use among PWH in the United States use have found that PWH have higher plasma inflammatory biomarkers such as sCD14, a marker of monocyte activation, establishing a link between higher frequencies of SU, inflammation, HIV, and mortality [68]. 

Although the emerging evidence in non-African populations suggests that these outcomes may be mediated by inflammation, there are no large-scale prospective cohort studies of PWH who use substances in SSA. Such studies could elucidate the mechanisms linking SU among PWH to disease progression and early mortality. Recent estimates suggest that only 1 in 16 Africans with a SUD (compared to 1 in 8 worldwide) has access to treatment. In South Africa, fewer than 5% of people who need SUD treatment are able to access it, and there are major disparities in both access to and quality of SUD services [69,70]. Among those accessing treatment, large geographical disparities exist; for example, in low- and middle- income countries (LMICs), programs to address SU and SUD do not exist or are significantly underfunded and cannot address the disease burden [21]. In addition, lack of treatment options for PWH in LMICs and the associated negative health outcomes from both HIV and SU produce a syndemic, which will result in increased mortality for PWH. 

The emergence of the coronavirus disease 2019 (COVID-19) has affected the supply chain for various legal substances and limited their availability at destination markets. Many countries in all regions have reported an overall shortage of numerous types of illicit substances at the retail level, as well as increases in prices and reductions in purity. Consequently, people who use drugs have been switching substances (for example, from heroin to synthetic opioids); and in LMICs, treatment programs have become more difficult to access [21]. At the time of writing, implementation of increased control measures to fight the spread of COVID-19 have resulted in some countries, such as Italy, Niger, and nations in Central Asia, experiencing a sharp decrease in drug seizures [71,72]. The results of the measures to curtail COVID-19 will be apparent only after the pandemic has subsided. We do not yet know how COVID-19 measures will have impacted drug distribution, trade, utilization, and PWH. Given that current data indicate that SU is increasing in Africa, the likely outcome is that more PWH will continue to use substances at higher levels compared to the general population, with accompanying increases in mortality and morbidity. 

## 6. Using a Holistic, Equity-Based Syndemics Framework for PWH with SU

While some societal forces that produce stigma and social exclusion are unique to each geographic, social, cultural, political, economic context in Nigeria, South Africa, and Uganda, the underlying disease pathways [73,74] (e.g., inflammatory mechanism) and non-disease pathways [75] (e.g., intersectional [76,77] and psychosocial [78,79,80]) that promote syndemic clustering of noncommunicable and communicable diseases for PWH are likely similar, based on context. The factors that drive syndemic emergence of disease for PWH in an LMIC are unique and different from the factors that drive emergence of disease in high income countries (HICs). For example, in a LMIC where TB and food insecurity are significant risks for PWH, those issues will play a much more significant role in intersectional stigma [77] of PWH, compared to sexual minority males living in a HIC, where food insecurity could be an issue but from a different contextual framework [81]. In both situations, SU may be a significant issue; however, to address the health outcomes of SU/SUD among PWH in LMICs, the customs, societal norms, general support structures of each group must be examined using an equity-based syndemics framework [82]. Essentially, this framework illustrates that various causal relationships cannot be disentangled from one another. A holistic approach is needed to address any syndemic—especially a multifaceted problem such as SU in PWH in SSA, where access to treatment is limited, food insecurity is common, and stigma is pervasive [83,84,85,86].

**Figure 1:**
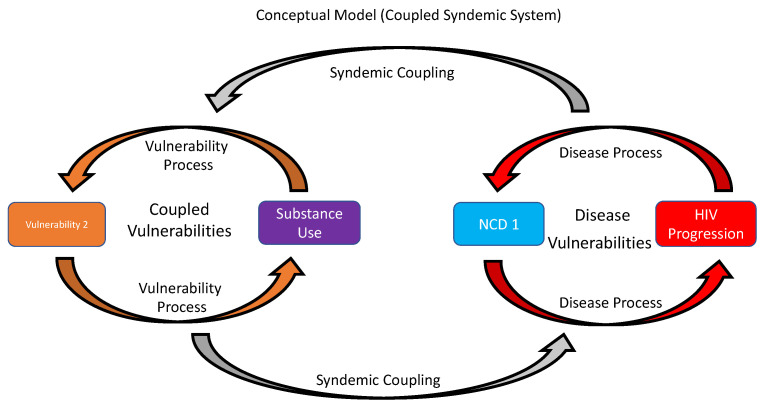
Syndemic coupling occurs when both a disease and a non-disease system interact as a coupled ecosystems that can amplify or attenuate HIV disease progression. The coupled vulnerabilities (orange arrows) include substance use and other vulnerabilities (e.g., poverty, intimate partner violence, etc.) interact in a vulnerability process can reinforce maladaptive behaviors. Vulnerability process (e.g., social, cognitive, socioeconomic, etc.) can couple with the disease process (red arrows) in that HIV progress can couple with non-communicable disease (e.g., HTN, and CVD) or another communicable disease (e.g., TB, Hepatitis B, etc.) and reinforce that disease processes. Both the disease process and vulnerabilities process are co-occurring and can either amplify or attenuate each system based on syndemic coupling (gray arrows).

In the equity-based syndemics framework, underlying disease mechanisms/pathways and non-disease pathways (e.g., intersectional and psychosocial stigma) can interact to produce a syndemic (Figure 1) that encompasses complex behavioral, physiological, and psychological pathways with overlapping vulnerability constructs, including both social and cognitive. Social vulnerabilities [87] are defined as abuse, social exclusion, etc., and can be correlated: HIV stigma [88,89] and cognitive vulnerabilities are thought to shape maladaptive processes and predispose the individual to psychological disorders [90]. Essentially the vulnerabilities (i.e., social and cognitive) in our conceptual model underlie behavioral pathways. Other vulnerabilities can also include social/community [91,92], socioeconomic [93], and various constructs. We coined the term “syndemic coupling” to describe the concept: co-occurring and mutually enhancing psychosocial and structural problems, as described by Tsai and Venkataramani [94]. In Figure 1, we depict both a disease and a non-disease system as coupled ecosystems that can amplify or attenuate HIV disease progression. Within this conceptual model, the coupled syndemic system contains two or more interdependent ecosystems, one biophysiological (e.g., HIV, TB, and cardiovascular disease) or any other disease and the other non-biophysiological (social/community, socioeconomic; e.g., stigma, and household income). Within each system, vulnerabilities have a dynamic cycle that can reinforce itself depending on forces outside the system (e.g., unemployment). We believe that like other systems, four different components contribute to syndemic coupling and define these systems: (1) the dynamics within one or more vulnerabilities; (2) the dynamics within one or more disease; (3) the processes through which the vulnerabilities affect the disease; and (4) the processes through which the disease affect the vulnerabilities. Coupled dynamic syndemic systems are, therefore, governed by vulnerabilities and human action and overlaid with biological and/or physical processes that include multilevel interactions, producing multidimensional feedback loops that interact at multiple levels with other factors at the individual, community, and society levels. For example, health disparities researchers have shown that adverse contextual and environmental circumstances can generate greater allostatic load, which, in turn, can affect and interact with risk factors to impact behavioral and lifestyle choices that ultimately influence and accelerate the development of disease [95,96,97]. Syndemic theory describes relationships between and among various syndemic factors that predispose PWH to disease clustering. Disentangling the dynamic interplay between coupled vulnerabilities, disease, and comorbid conditions among PWH will advance basic scientific understanding about how complex interactions of vulnerabilities produce syndemic coupling. The goal of syndemics research is to understand these factors and the role they have in disease progression. Moreover, there is an opportunity to consider malleable targets for interventions when assessing various relationships among syndemic factors so that clinical remedies can be designed to ameliorate disease. 

## 7. Conclusions

According to the UNODC, from 2018 to 2030 the number of drug users in Africa will increase by as much as 40%, to 86 million. This unprecedented transformation will challenge the cultural, socioeconomic, political, and health care environment of Africa and increase need for services to address SU. Moreover, it will be crucial to describe the biological underpinnings between HIV, inflammasome activation, inflammation, frequency of SU and SUD, and other clinical endpoints (e.g., stroke, CVD, and renal failure). Although some research has been conducted in HICs, some noteworthy differences between HICs and LMICs bring generalizability into question. These differences include cultural norms, health and socioeconomic disparities, and disparities in access to services that may worsen outcomes within African regions. Designing a multi-country cohort will provide key data and spur new research in this important, yet under-studied area of SU research for African populations living with HIV.

**Table 1:** Age-standardized estimates of substance use prevalence in sub-Saharan Africa per 100,000 people and DALYs attributable to alcohol and substances, Global Burden of Disease Study, 2016 [20,30,47,98].

	Central	Eastern	Southern	Western
Alcohol	1413.3	1611.0	1515.0	1168.1
Amphetamine	6.7	6.2	27.3	6.1
Cocaine	14.6	13.8	20.0	14.4
Opioids	240.1	212.1	376.8	276.4
Tobacco	179.1	230.9	189.0	116.8
Other substances	33.6	34.0	40.2	36.2
Alcohol DALYs (%)	2733.4 (2.9)	2010.6 (2.7)	3178.8 (5.1)	1166.0 (1.7)

**Table 2:** Age-standardized estimates of DALYs and mortality per 100,000 people attributable to alcohol and other substances (95% uncertainty interval), Global Burden of Disease Study, 2016, by country [20].

	Nigeria	South Africa	Uganda
Alcohol DALYs	1527.0 (922.6, 2257.0)	3012.6 (2409.0, 3610.2)	3694.5 (1945.1, 5565.9)
Drug DALYs	577.9 (467.8, 725.8)	572.0 (470.8, 705.0)	147.8 (113.5, 187.4)
Deaths attributable to alcohol	46.7 (23.0, 79.8)	88.5 (64.8, 110.1)	122.2 (56.6, 198.4)
Deaths attributable to substances	9.7 (7.6, 12.3)	376.7 (305.7, 502.1)	1.2 (0.9, 1.4)

## Data Availability

Not applicable.
